# Interdependence of domestic malaria prevention measures and mosquito-human interactions in urban Dar es Salaam, Tanzania

**DOI:** 10.1186/1475-2875-6-126

**Published:** 2007-09-19

**Authors:** Yvonne Geissbühler, Prosper Chaki, Basiliana Emidi, Nicodemus J Govella, Rudolf Shirima, Valeliana Mayagaya, Deo Mtasiwa, Hassan Mshinda, Ulrike Fillinger, Steven W Lindsay, Khadija Kannady, Marcia Caldas de Castro, Marcel Tanner, Gerry F Killeen

**Affiliations:** 1 Swiss Tropical Institute, Department of Public Health and Epidemiology, Socinstrasse 57, PO Box, 4002 Basel, Switzerland; 2 Ifakara Health Research and Development Centre, Co-ordination Office, Kiko Avenue, PO Box 78373, Dar es Salaam, Tanzania; 3 Dar es Salaam City Council, Dar es Salaam, Tanzania; 4 University of Dar es Salaam, Dar es Salaam, Tanzania; 5 School of Biological and Biomedical Sciences, South Road, Durham DH1 3LE, UK; 6 Department of Population and International Health, Harvard School of Public Health, 655 Huntington Avenue, Boston, MA 02115, USA

## Abstract

**Background:**

Successful malaria vector control depends on understanding behavioural interactions between mosquitoes and humans, which are highly setting-specific and may have characteristic features in urban environments. Here mosquito biting patterns in Dar es Salaam, Tanzania are examined and the protection against exposure to malaria transmission that is afforded to residents by using an insecticide-treated net (ITN) is estimated.

**Methods:**

Mosquito biting activity over the course of the night was estimated by human landing catch in 216 houses and 1,064 residents were interviewed to determine usage of protection measures and the proportion of each hour of the night spent sleeping indoors, awake indoors, and outdoors.

**Results:**

Hourly variations in biting activity by members of the *Anopheles gambiae *complex were consistent with classical reports but the proportion of these vectors caught outdoors in Dar es Salaam was almost double that of rural Tanzania. Overall, ITNs confer less protection against exophagic vectors in Dar es Salaam than in rural southern Tanzania (59% versus 70%). More alarmingly, a biting activity maximum that precedes 10 pm and much lower levels of ITN protection against exposure (38%) were observed for *Anopheles arabiensis*, a vector of modest importance locally, but which predominates transmission in large parts of Africa.

**Conclusion:**

In a situation of changing mosquito and human behaviour, ITNs may confer lower, but still useful, levels of personal protection which can be complemented by communal transmission suppression at high coverage. Mosquito-proofing houses appeared to be the intervention of choice amongst residents and further options for preventing outdoor transmission include larviciding and environmental management.

## Background

Malaria and other vector borne diseases are major contributors to the global burden of disease and a significant impediment to socioeconomic development in poor countries [1]. It is estimated that 300 to 660 million clinical attacks of malaria occur globally [2] which result in at least 1 million deaths [3,4]. Over 80% of these deaths occur in Africa [4]. Approximately 70% of clinical malaria attacks occur in sub-Saharan Africa with the vast bulk of the remainder occurring in south East Asia [4]. Sub-Saharan Africa has the highest incidence because ideal climatic conditions for transmission are exacerbated by some of the world's most efficient malaria vectors, such as *Anopheles gambiae*, *Anopheles arabiensis *and *Anopheles funestus *[5].

While the bulk of malaria research in Africa has focused on rural areas, the growing importance of urban settings is increasingly recognized [6-11]. Transmission intensity is generally lower in urban areas but it is estimated that, by the year 2030, more than 50% of the African population will live in towns and cities [12] so improved understanding and evidence-based strategies for controlling urban malaria are needed. Urban areas differ from rural settings in that exposure to transmission is typically lower and access to diagnosis, treatment and preventative measures is much better [6-11]. As recently elucidated using detailed transmission models [13-15], such lower exposure levels lead to a lower level of immunity in the population as a whole, as well as to higher prevalence, morbidity, mortality and infectiousness in older age groups [6-10,16]. Furthermore, the distribution of seasonal and permanent breeding sites is highly localized, leading to patchy, heterogeneous transmission at particularly fine spatial scales [7,17-21]. Malaria prevalence and incidence tends to be much higher for residents living close to major larval habitats [19,22-24]. This is because mosquitoes tend not to disperse far from the breeding sites as blood meal and aquatic habitat resources are in close proximity to each other [19,25-27]. This may even be true for water bodies which are not suitable for larval development but do act as oviposition sites [28], possibly resulting in the proportion of infectious mosquitoes increasing with the distance from their location of actual emergence [29]. Urban settings often have large areas with relatively good housing and relatively high coverage with personal protection measures such as ITNs, repellents and coils [11,30-35] with the potential to force changes in epidemiologically relevant behavioural patterns of vector mosquitoes [36-49].

*Anopheles gambiae *and its sibling species *An. arabiensis *are the most important vectors of malaria in most parts of Africa, where they readily adapt to urban ecosystems by ovipositing and developing in atypical larval habitats such as domestic containers and polluted water bodies [50-52]. Although these species are most commonly found in artificial larval habitats, even in rural areas, this is particularly the case in towns and cities [51-57]. Despite the enormous importance of these mosquito species, relatively little is known about their feeding behaviour, and even less about their broader ecology, particularly in urban setting. Furthermore, the influence of insecticide-treated nets (ITNs) [4,58,59], improved housing [60,61] and other personal protection [62-65] methods upon their feeding behaviour has been discussed qualitatively but has yet to be evaluated in quantitative terms. There is one example of Zimbabwe, where after eight years of insecticide spraying more *An. gambiae *sensu lato (s.l.) (as sibling species within this complex were not resolved in that study) were caught biting outdoors than indoors whereas before the intervention there was no difference [66,67]. In many places throughout Africa, a reduced indoor biting was reported due to ITNs and impregnated curtains [37,39,42,45,46,68-71] through a combination of increased mosquito mortality caused by their insecticidal properties and the reduction of mosquito house entry caused by their excito-repellent properties [49,72,73]. Indoor biting rates of malaria vectors can be reduced by improved housing, specifically mosquito-proof screening, closed eaves, ceilings and sealed frames for windows and doors [19,60,61,74-78] and some recent studies suggest changes in their biting patterns in response to personal or household protection measures [36,79,80]. However, only 20% (4/20) of the studies described in these papers have been carried out in urban areas so here the behavioural interactions between vector mosquitoes and their human hosts in the context of a large-scale integrated malaria control programme in Dar es Salaam, Tanzania are examined [52,81].

In Dar es Salaam, the main malaria vectors are members of the *An. gambiae *species complex and *An. funestus *[82]. Dar es Salaam has a relatively high coverage with bednets and ITNs (91.8% and 43.1%, respectively) [33]. In order to see if increasing ITN usage and house quality has influenced mosquito biting behaviour, a survey of behavioural interactions between mosquitoes and humans during the main rains of 2006 was undertaken. This study was also carried out in order to estimate the extent of protection against exposure to malaria transmission that is afforded to residents of Dar es Salaam by using an ITN and to evaluate the influence of housing quality upon this level of protection. Furthermore, the implication these behaviours have for malaria control in Dar es Salaam and elsewhere in Africa where similar trends are observed are discussed.

## Methods

### Study site

**Figure 1:**
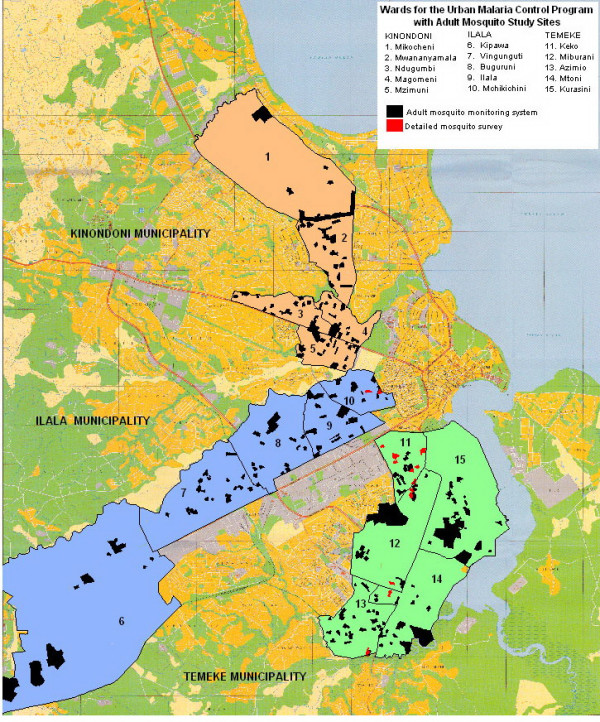
Wards included in the study area of the Urban Malaria Control Program in Dar es Salaam, showing the ten cell units (TCU) of the adult mosquito monitoring system as well as of the detailed survey.

Dar es Salaam is situated at the shores of the Indian Ocean coast with a hot and humid climate which is ideal for mosquito proliferation and malaria transmission, satisfying the climatic requirement for stable transmission of temperatures between 22°C and 32°C and a rainfall of around 80 mm per month for at least five months per year [83]. There are typically two rainy seasons: a main rainy season from March to June and a shorter, more erratic rainy season from October to December. Dar es Salaam has around 2.5 million inhabitants and covers a total area of 1,400 km^2 ^[84]. The city is divided into three municipalities; Temeke, Ilala and Kinondoni which collectively comprise 73 wards. Each ward is further subdivided into neighbourhoods known as *mitaa *(singular *mtaa*) which typically comprise between 20 and 100 *mashina *(singular *shina*) or Ten Cell Units (TCU). The TCU is the smallest subunit of local government in Tanzania which, in principle, comprises a cluster of 10 houses with an elected representative known as a *mjumbe *although in practice most TCUs include 20–30 houses and some may even exceed 100. This study was based within the project area of the ongoing Urban Malaria Control Programme (UMCP) implemented by the Dar es Salaam City Council [52,81]. The main project area includes five wards from each municipality with a total of 67 *mitaa*. Overall, this study area covers an area of 55 km^2 ^with a total population of 609,514 people [84]. The houses surveyed here were located in five wards, eight mitaa (Figure 1).

For comparison, the results obtained in Dar es Salaam are contrasted with those obtained with similar methodology in the Kilombero Valley, a rural setting with intense perennial malaria transmission in southern Tanzania [85].

### Preliminary survey of the overall study site

For the purposes of routine monitoring and programme management, the UMCP surveys mosquito biting densities at 268 locations (four in each *mtaa*), distributed across the study area every four weeks. Initial trials proved that existing trapping technologies were not sufficiently sensitive to monitor the low densities of *An. gambiae *which occur across the study area. Therefore, outdoor human landing catch (HLC) [86] has been implemented as the standard sampling tool for adult mosquitoes as an interim measure until a suitable alternative is proven practical, effective and affordable. Once every four weeks at each location, HLC is conducted from 6 pm to 6 am for 45 minutes of each hour, allowing 15 minute breaks for rest, hot drinks and snacks. All collected mosquitoes are identified morphologically to genus and, in the case of *Anopheles *to species complex level [87,88]. Members of the *Anopheles gambiae *species complex are further resolved to sibling species level by polymerase chain reaction (PCR) [89]. The sporozoite infection status of each mosquito was determined by enzyme-linked immunoabsorbent assay as previously described [90].

HLCs between April and December 2005 were used to identify the primary vectors of malaria in Dar es Salaam and to test for variation by location in the distribution of *An. gambiae *biting activity across the night. Members of the *An. gambiae *species complex were identified as the major malaria vectors in Dar es Salaam (See Results) so only these species were considered in the following analysis and study design. The influence of location as a determinant of *An. gambiae *biting habits was tested by treating TCU unique ID for each sampled site as a fixed factor in a logistic model with the proportion of mosquitoes caught during typical sleeping hours of city residents (10 pm to 6 am; see results) as the outcome variable. This data set was also used to identify sites with the highest densities of *An. gambiae s.l. *for the detailed and intensive mosquito behavioural surveys described below.

### Detailed surveys of mosquito biting behaviour

The 12 TCU in Temeke municipality and 2 TCU in Ilala municipality, which had the highest *An. gambiae s.l. *densities in the UMCP surveillance system, were selected for further, more detailed, surveys of the behavioural patterns of mosquitoes and humans. Informed consent was obtained from 216 houses in order to conduct HLC both indoors and outdoors. In each house, HLC was conducted for one night from 6 pm to 7 am as described above except that catchers switched between indoor and outdoor stations every hour in order to preclude biases resulting from variations in individual attractiveness [91-93]. These human landing catch surveys took place during 10 weeks of the main rainy season between April and June 2006. In order to estimate the biting rate for a full hour, total catches per hour were divided by 0.75.

### Interview surveys of human behaviour and domestic protection measures

A brief interview was conducted with all household members present at the time of the interview. They were asked where they usually eat dinner, where they stay after dinner before going to bed, what time they go to bed and what time they typically get out of bed in the morning. Furthermore, they were asked which preventive measures, such as bednets or insecticides, they use to avoid mosquito bites. The quality of their houses, i.e. the quality of screening and availability of ceiling boards was examined in each household. In order to verify the sleeping and resting behaviours reported by residents during interviews, also surveys were conducted based on direct observation by walking through these TCUs once every hour of the night and counting the number of people seen outdoors. Direct observation surveys were conducted for three nights in each TCU. Once validated by direct observation (see results), the questionnaire reports were used to estimate proportion of the inhabitants in each of the three behavioural compartments (outdoor, indoor awake, indoor asleep) at each hour of the night.

### Estimating the protective efficacy of ITNs in terms of reduced biting exposure

Data from the human and mosquito behavioural surveys described above were integrated to evaluate the interaction between them using an extension of a recently developed mathematical model [85]. EIR is the product of the biting rate experienced by humans exposed to a vector population and the sporozoite infection prevalence of that mosquito population [94]. The latter is only reduced by community-level impacts of malaria interventions [95,96] so here personal protection purely in terms of biting rates and the impact that protective measures such as ITNs have upon them were estimated. First B_u,t_, the mean biting rate experienced by an unprotected individual at each time of the night (t), based on the proportion of time spent outdoors multiplied by the outdoor biting rate at that time (B_o,t_) plus the proportion of that hour spent indoors multiplied by the indoor biting rate at that time (B_i,t_) was calculated. The main difference between this model and the one of Killeen *et al. *is that, because of the available information from the questionnaires, there was the possibility to divide the indoor compartment into being indoor but not asleep (and therefore not under a bednet) and being indoor and asleep (and, therefore, protected if using a bednet). The proportion of people sleeping or trying to sleep in bed and indoors (S_t_) is not the same as the proportion of people staying indoors asleep or not asleep (I_t_). If people are unprotected because they do not have a bednet, it only matters if they are indoors or outdoors and thus they experience the following biting rate:

B_u,t _= B_o,t _(1-I_t_) + B_i,t _I_t_

The number of bites experienced per night, or nightly biting rate, for an unprotected non-user (B_u_) can thus be calculated by summing the relevant biting rates for each hour:

Bu=∑t=124Bu,t
 MathType@MTEF@5@5@+=feaafiart1ev1aaatCvAUfKttLearuWrP9MDH5MBPbIqV92AaeXatLxBI9gBaebbnrfifHhDYfgasaacH8akY=wiFfYdH8Gipec8Eeeu0xXdbba9frFj0=OqFfea0dXdd9vqai=hGuQ8kuc9pgc9s8qqaq=dirpe0xb9q8qiLsFr0=vr0=vr0dc8meaabaqaciaacaGaaeqabaqabeGadaaakeaacqqGcbGqdaWgaaWcbaGaeeyDauhabeaakiabg2da9maaqahabaGaeeOqai0aaSbaaSqaaiabbwha1jabcYcaSiabbsha0bqabaaabaGaeeiDaqNaeyypa0JaeGymaedabaGaeGOmaiJaeGinaqdaniabggHiLdaaaa@3CDF@

Note that an unprotected individual is defined as someone lacking any net whereas a protected individual is defined as someone regularly using an effectively insecticidal net. The nightly biting rate of a protected individual (B_p_) based on the combined nightly profiles of mosquito biting rate (B_u,t_) over time (t), the protective efficacy of ITNs (P), which is assumed to be constant, and the behaviour of humans which results in fluctuating adherence of ITN users over the course of the night was modelled. As here a more detailed behavioural survey was taken into account, the nightly biting rate of a protected individual is calculated by multiplying the proportion of time spend outdoors at a certain time of the night by the outdoor biting rate at that time (B_o,t_) plus the proportion of that hour being indoors but not asleep (I_t _- S_t_) multiplied by the indoor biting rate during that hour (B_i,t_) plus the proportion of that time spent indoors being asleep under an ITN multiplied by the indoor biting rate at that hour (B_i,t_) times the proportion of bites which can not be prevented by an ITN (1-P), as measured in experimental hut trials [44,97,98]. The effective adherence to ITN use at a given time of the night was assumed to be equivalent to the proportion of people sleeping at that time (S_t_). This assumption allows us to express the overall effect of this interaction as follows:

Bp=∑t=124Bp,t=∑t=124[Bo,t (1−It)+Bi,t (It−St )+Bi,tSt (1−P)]
 MathType@MTEF@5@5@+=feaafiart1ev1aaatCvAUfKttLearuWrP9MDH5MBPbIqV92AaeXatLxBI9gBaebbnrfifHhDYfgasaacH8akY=wiFfYdH8Gipec8Eeeu0xXdbba9frFj0=OqFfea0dXdd9vqai=hGuQ8kuc9pgc9s8qqaq=dirpe0xb9q8qiLsFr0=vr0=vr0dc8meaabaqaciaacaGaaeqabaqabeGadaaakeaacqqGcbGqdaWgaaWcbaGaeeiCaahabeaakiabg2da9maaqahabaGaeeOqai0aaSbaaSqaaiabbchaWjabcYcaSiabbsha0bqabaaabaGaeeiDaqNaeyypa0JaeGymaedabaGaeGOmaiJaeGinaqdaniabggHiLdGccqGH9aqpdaaeWbqaaiabbUfaBjabbkeacnaaBaaaleaacqqGVbWBcqqGSaalcqqG0baDaeqaaOGaeeiiaaIaeeikaGIaeeymaedccaGae8NeI0IaeeysaK0aaSbaaSqaaiabbsha0bqabaGccqqGPaqkcqWFRaWkcqqGcbGqdaWgaaWcbaGaeeyAaKMaeeilaWIaeeiDaqhabeaakiabbccaGiabbIcaOiabbMeajnaaBaaaleaacqqG0baDaeqaaOGae8NeI0Iaee4uam1aaSbaaSqaaiabbsha0bqabaGccqqGGaaicqqGPaqkcqWFRaWkcqqGcbGqdaWgaaWcbaGaeeyAaKMaeeilaWIaeeiDaqhabeaakiabbofatnaaBaaaleaacqqG0baDaeqaaOGaeeiiaaIaeeikaGIaeeymaeJae8NeI0IaeeiuaaLaeeykaKIaeeyxa0faleaacqqG0baDcqGH9aqpcqaIXaqmaeaacqaIYaGmcqaI0aana0GaeyyeIuoaaaa@7163@

Based on existing evidence from experimental hut trials [49,97,98], a conservative minimum protective efficacy level of 80% for ITNs (P = 0.8), equivalent to a relative exposure to bites of 20% *when, and ****only when****, actually sleeping under the net*, was assumed. In this study, it was possible to take into account the proportion of people staying indoors or outdoors during waking hours and experiencing the corresponding biting rate. Furthermore, there was the possibility even to do the same for people living in different house quality who spent different amount of time in different compartments. During sleeping hours, people staying indoors were presumed sleeping under an ITN if available, whereas people sleeping outdoors were presumed not using a net and being fully exposed to the outdoor biting rate.

Taking the data for nightly human and mosquito behaviour profiles, the relative biting rate for ITN users which is equivalent to relative availability of protected individuals (λ_p_) as previously defined (See equations 8 and 14 in reference [95]), could be estimated. λ_p _was calculated by comparing the total biting rate that protected individuals are exposed to (B_p_) with that of non-users (B_u_) who are unprotected:

λ_p _= B_p_/B_u_

The *true *protective efficacy of an ITN (P*) against transmission exposure is then calculated as the overall nightly reduction of biting rate:

P* = 1 - λ_p_

This estimate of protective efficacy differs from that previously reported from experimental hut trials as well as previous applications of this approach [85], because it allows for typical shortcomings in adherence resulting from the time people typically spend outside of their ITN indoor, as well as outdoors and even considering people staying or sleeping the whole night outdoors. Note, however, that this estimate is merely a comparison between the biting rates experienced by those who use an ITN and those who do not. It does not include the community-level protection of both groups when ITNs reach sufficient levels of coverage to reduce vector biting densities and sporozoite prevalence over large areas [95].

Distinct and useful indicators with which to interpret the results of the above equations are the proportion of exposure which occur indoors and the proportion that occurs during sleeping hours. The proportion of bites that occur during the observed peak sleeping hours (π_s_) for an unprotected individual can thus be calculated as the nightly biting rate experienced during these hours divided by the total nightly biting rate:

πs=∑t=10pm6amBu,t/∑t=124Bu,t
 MathType@MTEF@5@5@+=feaafiart1ev1aaatCvAUfKttLearuWrP9MDH5MBPbIqV92AaeXatLxBI9gBaebbnrfifHhDYfgasaacH8akY=wiFfYdH8Gipec8Eeeu0xXdbba9frFj0=OqFfea0dXdd9vqai=hGuQ8kuc9pgc9s8qqaq=dirpe0xb9q8qiLsFr0=vr0=vr0dc8meaabaqaciaacaGaaeqabaqabeGadaaakeaacqaHapaCdaWgaaWcbaGaee4Camhabeaakiabg2da9maaqahabaGaeeOqai0aaSbaaSqaaiabbwha1jabcYcaSiabbsha0bqabaGccqGGVaWldaaeWbqaaiabbkeacnaaBaaaleaacqqG1bqDcqGGSaalcqqG0baDaeqaaaqaaiabbsha0jabg2da9iabigdaXaqaaiabikdaYiabisda0aqdcqGHris5aaWcbaGaeeiDaqNaeyypa0JaeGymaeJaeGimaaJaeeiCaaNaeeyBa0gabaGaeGOnayJaeeyyaeMaeeyBa0ganiabggHiLdaaaa@5075@

Note that π_s _describes the proportion of human exposure during which an ITN is in use and is used as a key parameter for modelling the community- and individual-level effects of ITNs upon malaria transmission [95]. Overall, π_s _was usually calculated using median reported values of 10 pm to 6 am for the whole study area but was evaluated separately for individual houses or houses with different quality of screening and ceiling boards for some analysis.

The proportion of bites occurring indoors but while awake and, therefore, not protected by a bednet (π_a_) can be calculated as the estimated number of bites estimated to occur indoors while awake, divided by the total number of bites estimated to occur both indoors and outdoors:

πa=∑t=124[Bi,t(It−St)]/∑t=124[Bo,t(1−It)+Bi,tIt]
 MathType@MTEF@5@5@+=feaafiart1ev1aaatCvAUfKttLearuWrP9MDH5MBPbIqV92AaeXatLxBI9gBaebbnrfifHhDYfgasaacH8akY=wiFfYdH8Gipec8Eeeu0xXdbba9frFj0=OqFfea0dXdd9vqai=hGuQ8kuc9pgc9s8qqaq=dirpe0xb9q8qiLsFr0=vr0=vr0dc8meaabaqaciaacaGaaeqabaqabeGadaaakeaacqaHapaCdaWgaaWcbaGaeeyyaegabeaakiabg2da9maaqahabaGaei4waSLaeeOqai0aaSbaaSqaaiabbMgaPjabcYcaSiabbsha0bqabaGccqGGOaakcqqGjbqsdaWgaaWcbaGaeeiDaqhabeaakiabgkHiTiabbofatnaaBaaaleaacqqG0baDaeqaaOGaeiykaKIaeiyxa0Laei4la8YaaabCaeaacqGGBbWwcqqGcbGqdaWgaaWcbaGaee4Ba8MaeiilaWIaeeiDaqhabeaaaeaacqqG0baDcqGH9aqpcqaIXaqmaeaacqaIYaGmcqaI0aana0GaeyyeIuoakiabcIcaOiabigdaXiabgkHiTiabbMeajnaaBaaaleaacqqG0baDaeqaaOGaeiykaKIaey4kaSIaeeOqai0aaSbaaSqaaiabbMgaPjabcYcaSiabbsha0bqabaaabaGaeeiDaqNaeyypa0JaeGymaedabaGaeGOmaiJaeGinaqdaniabggHiLdGccqqGjbqsdaWgaaWcbaGaeeiDaqhabeaakiabc2faDbaa@66BF@

The proportion of bites occurring indoors (π_i_) for an unprotected individual can be calculated as the total number of bites estimated to occur indoors, divided by the total number of bites estimated to occur both indoors and outdoors. It should be noted that this equivalent to summing π_a _and π_s_:

πi=πa+πs=∑t=124[Bi,tIt]/∑t=124[Bo,t(1−It)+Bi,tIt]
 MathType@MTEF@5@5@+=feaafiart1ev1aaatCvAUfKttLearuWrP9MDH5MBPbIqV92AaeXatLxBI9gBaebbnrfifHhDYfgasaacH8akY=wiFfYdH8Gipec8Eeeu0xXdbba9frFj0=OqFfea0dXdd9vqai=hGuQ8kuc9pgc9s8qqaq=dirpe0xb9q8qiLsFr0=vr0=vr0dc8meaabaqaciaacaGaaeqabaqabeGadaaakeaacqaHapaCdaWgaaWcbaGaeeyAaKgabeaakiabg2da9iabec8aWnaaBaaaleaacqqGHbqyaeqaaOGaey4kaSIaeqiWda3aaSbaaSqaaiabbohaZbqabaGccqGH9aqpdaaeWbqaaiabcUfaBjabbkeacnaaBaaaleaacqqGPbqAcqGGSaalcqqG0baDaeqaaOGaeeysaK0aaSbaaSqaaiabbsha0bqabaGccqGGDbqxaSqaaiabbsha0jabg2da9iabigdaXaqaaiabikdaYiabisda0aqdcqGHris5aOGaei4la8YaaabCaeaacqGGBbWwcqqGcbGqdaWgaaWcbaGaee4Ba8MaeiilaWIaeeiDaqhabeaakiabcIcaOiabigdaXiabgkHiTiabbMeajnaaBaaaleaacqqG0baDaeqaaOGaeiykaKIaey4kaSIaeeOqai0aaSbaaSqaaiabbMgaPjabcYcaSiabbsha0bqabaGccqqGjbqsdaWgaaWcbaGaeeiDaqhabeaakiabc2faDbWcbaGaeeiDaqNaeyypa0JaeGymaedabaGaeGOmaiJaeGinaqdaniabggHiLdaaaa@6A02@

### Ethical considerations

All activities of the UMCP, including these field surveys are approved by the Medical Research Coordination Committee of the National Institute for Medical Research, Ministry of Health, Government of Tanzania (Reference numbers NIMR/HQ/R.8a/Vol. IX/279 and 324). No persons in high risk groups, namely people under 18 years or women of reproductive age, were recruited to conduct human landing catches. Furthermore, the human landing catchers were screened every week for malaria microscopic examination of thick smear peripheral blood samples and treated with artemisinin-based combination therapy when diagnosis was positive.

## Results and Discussion

### Preliminary surveys of the entire study area

In the areas in Dar es Salaam which were covered by the urban malaria control programme (UMCP) during the first three rounds of the household surveys, bed net usage was quite high and mosquito-proofed houses were common with many being made of concrete or bricks with a corrugated iron roof (Table 1). Around half of the houses had a complete ceiling board and/or good screening although a small proportion of residents didn't use any protection measures at all. The same was true in the TCUs which were selected for the more detailed study (Table 2). When compared to historical reports from Dar es Salaam, bednet usage had increased whereas the use of other protective measures had decreased [34]. In contrast, in the Kilombero Valley in southern Tanzania, where ITNs have been promoted since 1997, bednet use is currently approximately at the same level, but both treatment of these nets and the use of other protective measures (coil, spray or repellent) are higher in Dar es Salaam (Killeen et al, Unpublished). Bed net usage in two contemporary Kenyan cities in 2001 was slightly lower and it should be noted that while screening of houses was less common than in Dar es Salaam, use of personal protection measures was more common [99].

**Table 1:** Characteristics of the houses and residents in all 15 wards of the study area in Dar es Salaam, Tanzania, during the first three rounds of household surveys from May 2004 until May 2006

Characteristic	Frequency	
	
	N	%
***Houses***	***3073***	***100***
*Walls*	3073	100
Stone, cement, fired or concrete bricks	1684	54.4
Unfired bricks, sand, wood	1355	43.7
Corrugated iron sheets, mud, grass	59	1.9
Grass thatch, cardboard	0	0
		
*Roof*	*3073*	*100*
Tiles, cement, reinforced concrete	193	6.3
Corrugated iron sheets, asbestos	2868	93.3
Thatch, sticks, mud, grass, plastic sheets	11	0.4
		
*Ceiling board*	*3066*	*100*
Whole house	829	27
Partly	554	18.1
None	1683	54.9
		
*Screening*	*3057*	*100*
Intact	684	22.4
With holes	1006	32.9
Incomplete	503	16.5
Glass windows	105	3.4
None	759	24.8
		
***Residents***	***20289***	***100***
*Bednet coverage*	20285	100
User	16883	83.2
Non-user	3402	16.8
		
*Treatment status of net*	16883	100
Treated in last 6 months	5194	30.8
Treated more than 6 months ago	66	0.4
Never treated	11623	68.8
		
*Other protection against mosquitoes*	20287	100
Coil	1245	6.1
Spray	2167	10.7
Repellent	307	1.5
None	16571	81.7
		
*Usage of at least 1 protection measure*	20289	100
Net, coil, spray, repellent	17437	85.9
None	2852	14.1

**Table 2:** Protection measures against mosquitoes in urban Dar es Salaam in the past and present, in rural Tanzania and in two Kenyan cities

**Location**	**Net usage**		**Net treatment status**				**Window screening**				**Other protection measures**			
	%	N	In the last 6 months	More than 6 months	never	intact	Small holes	Big holes	Glass windows	none	Coil	repellent	spray	none

*Urban Kenya and Tanzania*														
Dar es Salaam; 2006^a^	78.8	1696	35.9	0.6	63.5	17.3	39.6	14.7	0.4	28	9.2	7.3	15.9	67.6
Dar es Salaam; 1994 [34]	62										52	-	30	18
												
Kisumu, Kenya; 2001 [99]	56	287				5				95	40	0.008	13	47
Malindi, Kenya; 2001 [99]	69	332				32				68	54	27	5	14
														
*Rural Reference Site*														
														
Kilombero, Valley Tanzania;2003 (Killeen et al. unpublished)	74.5	650	4.7	6.6	88.7								1.4^b^	98.6

^a ^Data derived from the study presented here.

^b ^Any type of protection measures (spray, coil, herbal, physical)

**Figure 2:**
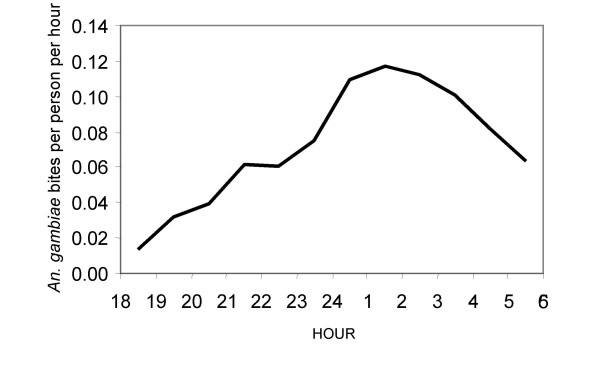
Hourly biting profile of *An. gambiae s.l. *based on averaged results of routine outdoor human landing catches from across the entire study area covered by the Urban Malaria Control Programme.

A total of 1,388 *An. gambiae *s.l. (meaning members of the species complex as a whole in the absence of further identification to species by cytological or molecular methods) were caught in 1,650 catcher-nights, through routine monitoring activities of the UMCP during the preliminary survey of the entire study area (Figure 2). The majority of these proved to be *An. gambiae *(often referred to as *An. gambiae sensu stricto*): 75.6%, 21.3% and 3.1% of 1099 successfully amplified specimens proved to be *An. gambiae s.s.*, *An. arabiensis *and *Anopheles merus*, respectively. During the same preliminary surveys, only 55 *An. funestus *were caught, indicating that although it is usually a very efficient vector [87], its contribution to transmission in urban Dar es Salaam is minor. Nevertheless, sporozoite infection and local transmission within urban Dar es Salaam was confirmed for *An. gambiae s.s. *(0.24%; 2/831) and *An. funestus *(2.32%, 1/43), but not *An. arabiensis *(0.0%, 0/234) and *An. merus *(0.0%, 0/34). Estimates of actual transmission intensity and its spatio-temporal heterogeneity over longer, more representative time periods will be reported in detail elsewhere. The only other *Anopheles *species caught was *Anopheles coustani *(370), of which none were found to be sporozoite-infected, so it is thought to contribute little or no vectorial capacity as described elsewhere [87].

*Anopheles gambiae s.s. *was by far the most important vector in the study area so all subsequent analysis focus upon this species and, to a lesser extent, *An. arabiensis*. Based on preliminary surveys of the total study area, location had no influence upon the proportion of *An. gambiae s.l. *bites which occurred between 10 pm and 6 am when residents of Dar es Salaam typically slept (*An. gambiae s.s.*: P = 0.519 by logistic regression, N = 72 locations, n = 714 mosquitoes, *An. arabiensis*: P = 0.398 by logistic regression, N = 32 locations, n = 133 mosquitoes). The great majority of the combined bites of these species occurred during sleeping hours (π_s _= 83.16%; equation **6**). Subsequent detailed surveys of mosquito and human behaviours therefore focussed upon the 14 TCUs with the highest *An. gambiae *densities observed during the preliminary site-wide surveys (Figure 1).

### Detailed focal surveys of household and personal protection

A total of 2,153 people were living in these 216 houses at the time of survey, of whom approximately half were under the age of 22 (Table 3). All the TCU were either near a swamp or close to a depression with poorly functioning drains and most of these areas were partially flooded during the rains. Although these were mostly poorer, unplanned areas, half of the houses had intact screening or screening with small holes. Almost three quarters of these houses did not have a ceiling board and it was typically observed that the eaves of most houses in Dar es Salaam were accessible to mosquitoes. Although more than three quarters of residents slept under a net, only a third of these nets had ever been treated with insecticide. Very few residents reported using alternative protective measures such as repellents, mosquito coils or insecticidal sprays (Table 3).

**Table 3:** House characteristics and human behaviour traits (time period from February to June 2006) of the areas in Dar es Salaam where mosquitoes were sampled indoors and outdoors

Characteristic	Frequency	
	
	N	%
*Age*		
<1 year	62	2.9
1–5 years	231	10.7
6–14 years	403	18.7
>14 years	1457	67.7
*Ceiling board*		
Whole house	37	16.6
Partly	27	12.1
None	159	71.3
*Screening*		
Intact	44	19.7
With holes	90	40.4
Incomplete	31	13.9
Glass windows	2	0.9
None	56	25.1
*Bednet usage*		
Overall	1695	78.8
<1 year	53	96.4
1–5 years	213	92.6
6–14 years	322	79.9
>14 years	1107	76
*Treatment status of net*		
Treated in last 6 months	774	35.9
Treated more than 6 months ago	11	0.6
Never treated	1368	63.5
*Other protection against mosquitoes*		
Coil	198	9.2
Spray	343	15.9
Repellent	158	7.3
None	1454	67.6
*Eating location*		
Indoor	783	74.1
Outdoor	270	25.6
Other	3	0.3
*Dinner time*		
Before 7 pm	59	5.6
Between 7 and 8.30 pm	492	46.6
After 8.30 pm	505	47.8
*Resting location after dinner*		
Indoor	505	47.8
Outdoor	540	51.1
Other or don't know	11	1.1
*Bedtime*		
Before 6 pm	3	0.3
Between 6 and 7 pm	18	1.7
Between 7 and 8 pm	48	4.5
Between 8 and 9 pm	117	11.1
Between 9 and 10 pm	312	29.5
Between 10 and 11 pm	379	35.9
Between 11 and 12 pm	125	11.8
After 12 pm	53	5
Don't know	1	0.1
*Waking time*		
Before 4 am	4	0.4
Between 4 and 5 am	23	2.2
Between 5 and 6 am	173	16.4
Between 6 and 7 am	509	48.2
After 7 am	346	32.8
Don't know/didn't respond	1	0.1
*Sleeping location*		
Outdoor sleeping	56	5.3
Indoor sleeping	1000	94.7

### Human-mosquito behavioural interactions

**Figure 3:**
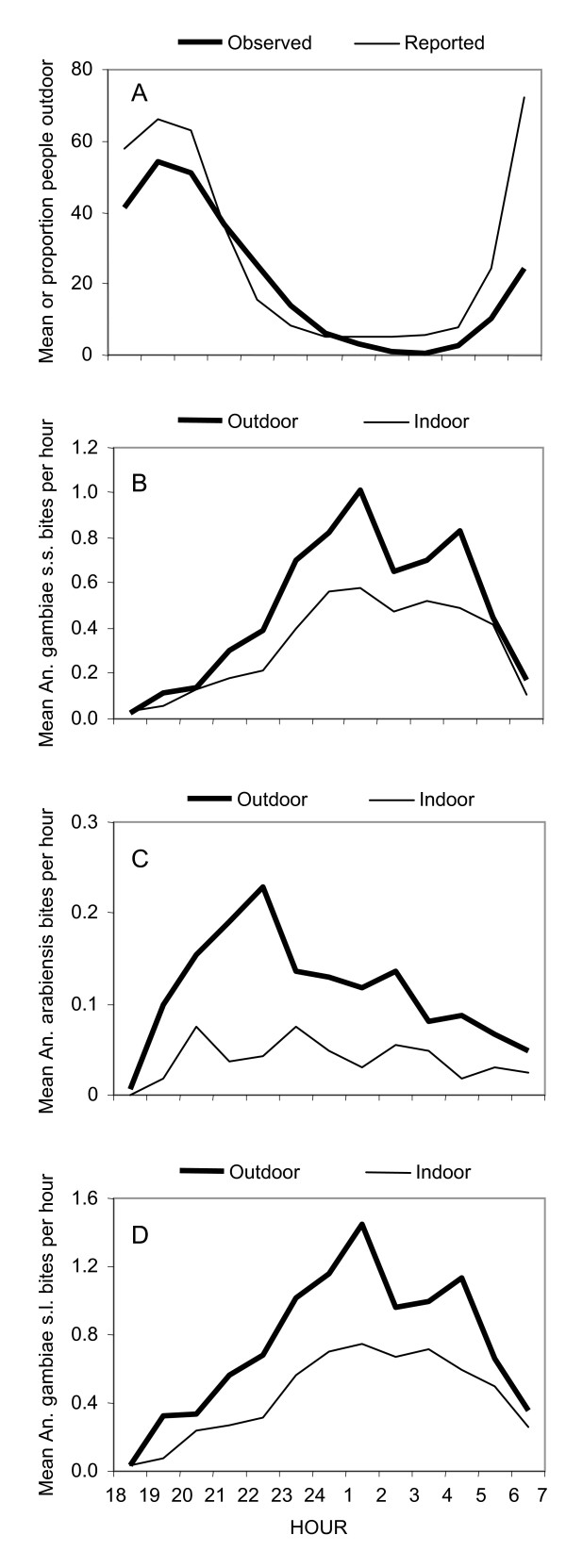
Human and mosquito behavioural patterns in Dar es Salaam, Tanzania. **A**. Number or proportion of time residents spend outdoors, comparing what they reported themselves with direct observations in the field. **B**. Mean numbers of *An. gambiae s.s *caught indoors and outdoors. **C**. Mean number of *An. arabiensis *caught indoors and outdoors. **D**. Mean number of *An. gambiae s.l. *caught indoors and outdoors.

The reported and observed behaviours of humans were largely consistent (Figure 3A). The minor discrepancies can be explained as follows. Less people were observed than reported outdoors in the evenings and mornings, because it was not possible for us to enter all courtyards and some individuals may be elsewhere during these hours. More people were observed than reported to be outdoors towards midnight but, based on direct experience, this was attributed to the transition of people through the TCU who do not live there. The residents reported that shortly after 10 pm, 50% of the people had gone to bed and at around 6 am 50% of the people were still asleep. A small, but noteworthy, proportion of residents slept outdoors all night (Table 3), often citing heat and poor ventilation inside the house as their primary motivation.

**Figure 4:**
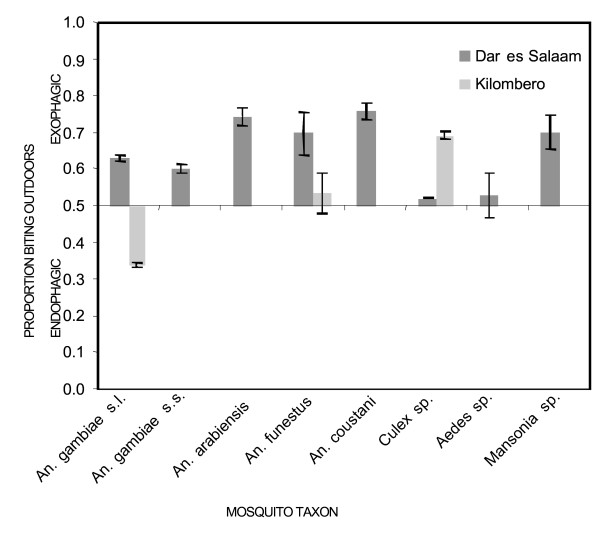
Comparison of exophagic and endophagic behaviour of different mosquito species in urban and rural Tanzania. Degree of exophagy or endophagy is presented as the proportion of mosquitoes caught outdoors so that all mosquitoes with a proportion of outdoor biting significantly greater than 0.5 are considered to be exophagic and all below 0.5 are considered endophagic.

During the intensive entomological study in the selected sites with high *An. gambiae *densities, 432 catcher-nights yielded 2,484 *An. gambiae *s.l., 63 *An. funestus*, 370 *An. coustani*, 41,290 *Culex*, 70 *Aedes *and 97 *Mansonia*. Of the 2,027 *An. gambiae s.l. *which were successfully amplified, 83.9%, 15.9% and 0.2% were identified as *An. gambiae s.s.*, *An. arabiensis *and *An. merus*, respectively. Only 0.41% (7/1700) of *An. gambiae s.s. *and 0.31% (1/322) and *An. arabiensis *were found to be infected with sporozoites. *An. gambiae *s.s., *An arabiensis*, *An. funestus*, *An. coustani *and *Mansonia *were all exophagic, meaning that they mainly bite outdoors [100] as evidenced by the proportion of mosquitoes caught outside being significantly greater than half (Figures 3 and 4). *Anopheles gambiae s.l. *is generally endophagic in rural Tanzania [36,87,101] and the proportion of *An. gambiae *s.l. caught outdoors was higher in Dar es Salaam than in Kilombero valley (Figure 4; 63 versus 34%, respectively; Χ^2 ^= 597.1, P < 0.001), considering only catches up to 6 am because the studies in Kilombero valley stopped at this time. In Dar es Salaam, the proportion of *An. arabiensis *caught outdoors was significantly higher than the proportion of *An. gambiae s.s. *caught outdoors (Χ^2 ^= 23.4, P-value < 0.001). *Culex *sp. and *Aedes *sp. exhibited neither exo- nor endophagic tendencies in Dar es Salaam.

Hourly biting pattern almost exactly followed classically reported patterns of *An. gambiae s.l. *[87] with an increase of *Anopheles gambiae s.s. *densities towards midnight, and a second peak around 4 – 5 am, followed by a decline towards dawn (Figure 3B). In fact, the proportions of *An. gambiae s.s *mosquitoes caught during peak sleeping hours was greater in the city than in the rural area (Χ^2 ^= 112.9, P < 0.001) with peak sleeping hours in Kilombero valley from 9 pm to 5 am and in Dar es Salaam from 10 pm to 6 am. As summarized in Figure 4, biting activity was more intense outdoors than indoors throughout the night and was highest during sleeping hours (Figure 3B). *An. gambiae s.s. *constituted 84% of *An. gambiae s.l. *and therefore dominates the shape of the curve for the pooled sibling species (Figure 3D). Nevertheless, it is noteworthy that *An. arabiensis *had its peak biting time at 10 pm, when more than three quarters of the residents were still awake, and then slowly declined towards the morning (Figure 3C).

**Figure 5:**
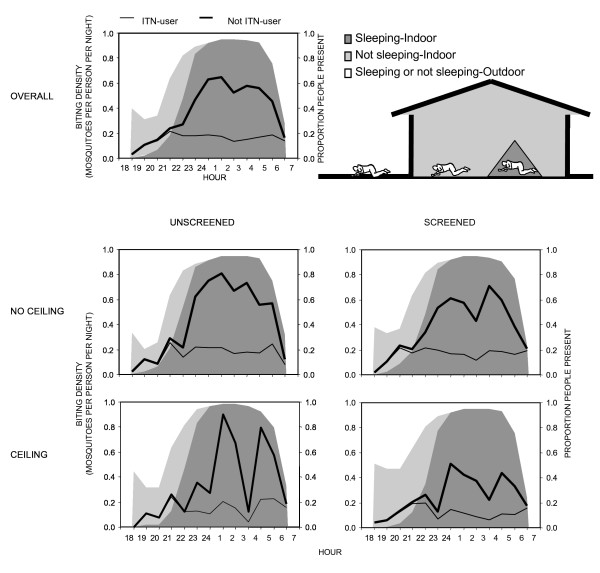
Exposure to biting of *An. gambiae s.s. *for ITN users and non-users. The shadings represent the proportion of time spend in each compartment (outdoor; 1-π_i _; equation **8**, indoor awake; π_a _; equation **7**, indoor asleep; π_s _; equation **6**). Exposure to biting is shown overall as well as for different house qualities: Screened (Glass windows, screening with no or small holes), unscreened (no screening or badly torn/incomplete screens), ceiling (complete ceiling or partly ceiling), no ceiling (no ceiling board).

**Figure 6:**
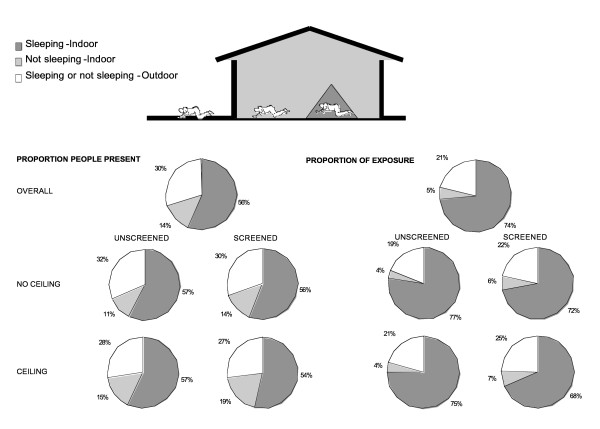
Proportion of people present in each compartment and their estimated exposure if not using a bednet (outdoor; 1-π_i _; equation **8**, indoor awake; π_a _; equation **7**, indoor asleep; π_s _; equation **6**), presented as an overall mean and for categories of different house qualities: Screened (Glass windows, screening with no or small holes), unscreened (no screening or badly torn), ceiling (complete ceiling or partly ceiling), no ceiling (no ceiling board).

Combining the human and mosquito behavioural surveys, and using the model described in the methods section, allowed estimation of the biting rates experienced by residents at each hour of the night (Figure 5). This approach also allowed dissection of these mosquito-human interactions into distinct domestic compartments (Figure 6) where specific interventions may or may not reduce exposure. For example, ITNs are expected only to provide personal protection while sleeping so their protective efficacy is limited to those times of the night when users sleep and cannot exceed the proportion of exposure which would otherwise occur while asleep (π_s_; equation **6**). In contrast, interventions which prevent house entry, such as mosquito proofing [60,61] or spatial repellents such as DDT [100], could prevent any indoor exposure regardless of whether occupants are awake or in bed (π_i_; equation **8**). It should be noted that the simpler form of this approach applied previously [85] did not allow estimation of exposure indoors while awake so it is not possible to compare Dar es Salaam with this rural precedent in terms of the relative contributions of exposure indoors and outdoors while awake. Nevertheless, it is possible to compare the proportion of exposure which an ITN might be expected to prevent (π_s_; equation **6**).

Even though *An. gambiae s.s. *were exophagic in urban Dar es Salaam, a high quality ITN was expected to confer 59% protection against exposure to this mosquito for a typical resident in a typical house. Although such protection against exposure is clearly incomplete, it is almost as high as the 70% protection afforded against highly endophagic *An. gambiae *in rural Kilombero [85] which is known to provide effective protection against clinical disease even in this highly endemic rural setting [102,103]. This slightly lower level of protection against exposure is because the number of bites which normally occur indoors and during sleeping hours were lower in the city (79% and 74%, respectively) than in the rural area (90% and 80%, respectively). The less abundant *An. arabiensis *was not only exophagic in Dar es Salaam but also most active just before 10 pm (Figure 3C) so the personal protection by an ITN against exposure to this species is estimated to be only 38%.

### Interdependence of protection measures and mosquito densities

**Figure 7:**
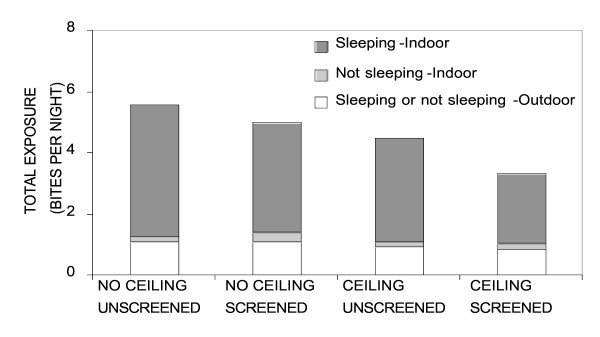
Mean number of bites received by a person in each of the three domestic and peri-domestic compartments (outdoor; 1-π_i _; equation **8**, indoor awake; π_a _; equation **7**, indoor asleep; π_s _; equation **6**).

Members of the *An. gambiae s.l. *complex dominated malaria transmission in Dar es Salaam and, of these, only *An. gambiae s.s. *was present in sufficient numbers to undertake the following analysis in a meaningful way. The following results only describe those for *An. gambiae s.s.*, as confirmed by PCR, and assume it is responsible for essentially all transmission in the study area. In well-screened (glass windows, screening with no or small holes) and houses with complete ceiling boards (complete and partly ceiling board) ITNs conferred slightly less protection against *An. gambiae s.s. *because the proportion (Figures 5 and 6) and total (Figure 7) levels of exposure in such houses that occurred indoors were lower. It should be noted that much of the reduction of proportional and total exposure achieved with screening and ceilings resulted from adaptive changes in human behaviour with occupants spending more of their waking hours in the safer confines of the house (Figures 6 and 7).

Exploratory pair-wise correlation analysis showed that complete ceilings were associated with use of other protection methods (r^2 ^= 0.323, P < 0.01) and good house screening (r^2 ^= 0.267, P < 0.01), which was in turn associated with high outdoor densities of *Culex *sp (r^2 ^= 0.136, P < 0.05). Interestingly, use of ITNs was associated with high indoor densities of *Culex *sp (r^2 ^= 0.137, P < 0.05) and use of any bednet was negatively correlated with complete ceilings (r^2 ^= -0.194, P < 0.01) and other protection methods (r^2 ^= -0.209, P < 0.01). This suggests that installation and maintenance of ceilings and screening, is motivated by local densities of nuisance mosquitoes whereas use of bednets may be a response to the failure or inability to apply these for socioeconomic reasons. The overall biting densities of *An. gambiae *showed only a negative association with complete ceilings (r^2 ^= -0.160, P < 0.05) and good screening (r^2 ^= -0.136, P < 0.05), suggesting that this vector species contributes little to motivating their utilization. Also, consistent with their known preference for eave entry and the results presented in figures 6 and 7, ceilings do confer protection against exposure to malaria transmission as does, to a lesser extent, good screening.

**Figure 8:**
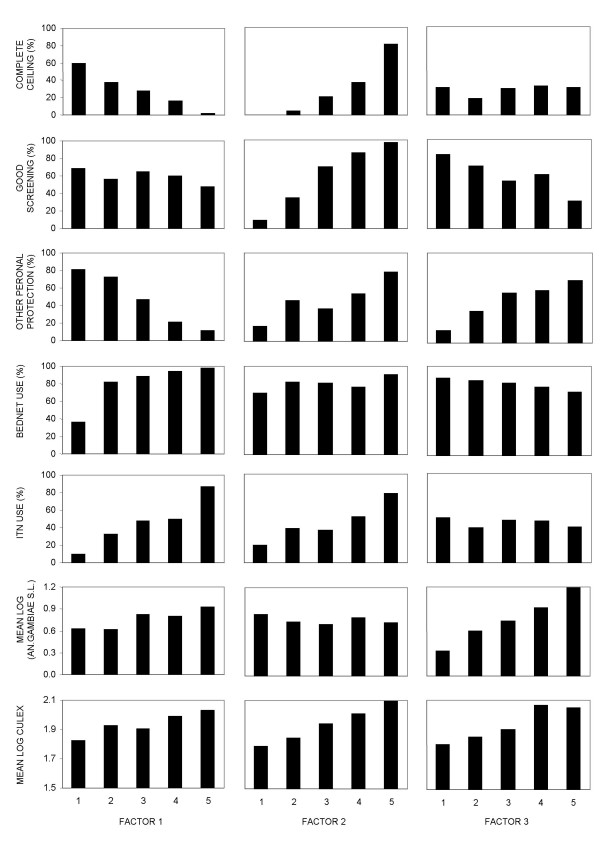
Three factors derived through principal component analysis and their association with different protective measures as well as mosquito densities.

Principal component analysis of the relationship between vector densities and the various protection measures surveyed revealed three important factors (Table 4), suggesting that the uptake and use of these interventions is driven by a number of motivations and constraints in a complex manner (Figure 8). Interestingly, Factor 2 shows clear increase in use of all protective measures associated with increased density of *Culex *sp. but not *An. gambiae s.l.*, probably reflecting the motivation for uptake of all interventions at high densities of nuisance biting. Factors 1 and 3 seem to reflect quite different underlying motivations or limitations that determine intervention utilization at household level and interact to a greater or lesser extent with mosquito density. Factor 1 shows a clear association of mosquito proofed houses with low usage rates of treated or untreated bednets and with high usage rates of other protective measures. This maybe reflects the influence of socioeconomic status on the choices of interventions used by households with mosquito-proofing and other measures probably being associated with better households while bednets may be utilized to a greater extent in houses which cannot afford these. Factor 3 appears to be almost completely independent of bednet use, but exhibits a clear association of the use of other interventions with high densities of *An. gambiae s.l. *and poor or absent window screening. It is suggested that factor three reflects the response of residents to indoor exposure to *An. gambiae*, perhaps as a proxy for malaria transmission, when window screening is not present. However these suggestions have to be looked at with caution as they remain speculative until such surveys of practice are conducted on larger population scales and complemented with direct evaluations of socioeconomic and educational status, as well as associated knowledge and attitudes.

**Table 4:** Protective measures, malaria and nuisance mosquito densities and their scores in three different factors and the percent of the variance these factors account for derived through principal component analysis.

		Factor 1	Factor 2	Factor 3
	% of Variance	24.55	21.41	15.67
Scores	Complete ceiling^a^	-0.477	0.646	0.039
	Good screening^b^	-0.155	0.629	-0.362
	Other personal protection^c^	-0.528	0.392	0.374
	Bednet use	0.773	0.236	-0.223
	ITN use	0.628	0.508	-0.076
	Mean log (*An. gambiae s.l*.)	0.334	-0.068	0.764
	Mean log Culex	0.289	0.463	0.430

^a ^Complete and partly complete ceiling board

^b ^Screening without holes, with small holes or glass windows

^c ^Coils, spray and/or repellent

## Conclusion

Although the hourly biting pattern of *An. gambiae s.s. *remains essentially consistent with classical reports, *An. arabiensis *appears to have a much earlier peak biting time at 10 pm when a large proportion of people are still outdoors. ITNs confer little protection against exposure to this species, which is fortunately relatively rare in urban Dar es Salaam. *Anopheles arabiensis *only account for 16% of the *An. gambiae *complex in Dar es Salaam, so ITNs still provide useful individual protection. However, the observations from Dar es Salaam can have greater implications for malaria control in Africa where *An. arabiensis *is a very common and an important vector [5,88,104]. It cannot be determined whether the early biting of *An. arabiensis *in Dar es Salaam was induced by ITN use and/or improved housing quality. In this context, it seems relevant to note that this *An. arabiensis *is more tolerant to desiccation than *An. gambiae *[88,105,106] and may, therefore, be able to adapt more readily to earlier feeding despite the relatively low humidity that occurs in the early evening. The surprisingly exophagic behavior of *An. gambiae *in Dar es Salaam may also arise from increased bednet coverage as well as housing quality. This is consistent with another recently reported urban context [80] and an increasing number of sites in rural Africa [107-111].

Despite the clear exophagy of malaria vectors in Dar es Salaam, like elsewhere in Africa, ITNs confer useful but incomplete personal protection [59,112]. Much bigger reductions of transmission can be attained at community level where high population coverage is achieved [44,95,113,114]. Although additional vector control measures are desirable to cope with the remaining quarter of human exposure which occurs outdoors, ITNs should remain a high priority in urban settings. ITNs appear to be a second preference intervention in Dar es Salaam, with mosquito-proofing of houses being the most commonly implemented measure and probably the first choice of residents. It may, therefore, be feasible to develop programmes which promote and subsidize such efforts by vulnerable households to tackle their local malaria problems. Additional important options to prevent outdoor transmission include larviciding [115,116] and environmental management [117-119], all of which merit further development as components of integrated programmes [1] in the tropical belt of Africa, where malaria transmission is at its most intense [5].

## Competing interests

Part of the Urban Malaria Control Programme is financed by Valent Biosciences Corporation, a manufacturer of microbial larvicides. A substantial portion of the current salary and research support for the investigators depends on the achievement of documented suppression of malaria transmission and infection risk by this programme through systematic larviciding.

## Authors' contributions

YG designed and implemented the study, analysed the data and drafted the manuscript. PC, BE, NJG, RS, VM, DM, HM, UF, SWL and KK were involved in designing and implementation the study. MCdC, MT and GFK participated in the study design, data analysis and drafting of the manuscript. All authors read and approved the final manuscript.
